# Bacterial autoaggregation

**DOI:** 10.3934/microbiol.2018.1.140

**Published:** 2018-03-01

**Authors:** Thomas Trunk, Hawzeen S. Khalil, Jack C. Leo

**Affiliations:** Bacterial Cell Surface Group, Section for Genetics and Evolutionary Biology, Department of Biosciences, University of Oslo, Oslo, Norway

**Keywords:** autoaggregation, autoagglutination, bacterial stress responses, biofilm, flocculation, microcolony formation, self-recognition

## Abstract

Many bacteria, both environmental and pathogenic, exhibit the property of autoaggregation. In autoaggregation (sometimes also called autoagglutination or flocculation), bacteria of the same type form multicellular clumps that eventually settle at the bottom of culture tubes. Autoaggregation is generally mediated by self-recognising surface structures, such as proteins and exopolysaccharides, which we term collectively as autoagglutinins. Although a widespread phenomenon, in most cases the function of autoaggregation is poorly understood, though there is evidence to show that aggregating bacteria are protected from environmental stresses or host responses. Autoaggregation is also often among the first steps in forming biofilms. Here, we review the current knowledge on autoaggregation, the role of autoaggregation in biofilm formation and pathogenesis, and molecular mechanisms leading to aggregation using specific examples.

## 1. Introduction

### 1.1. Bacterial autoaggregation as a phenomenon

In addition to adhering to host cells, the extracellular matrix of host tissues, or inorganic surfaces, many bacteria also have the ability to bind to themselves. This self-binding is termed autoaggregation or autoagglutination, and is along with surface colonization among the first steps in the formation of biofilm [1],[2]. Autoaggregation is macroscopically observed as the formation of bacterial clumps that settle at the bottom of culture tubes. In autoaggregation, bacteria of the same type, e.g. in pure culture, form these clumps. This is in contrast to co-aggregation, where bacteria of different strains or even different species associate [3]–[5]. Thus, autoaggregation can be regarded as a kind of self-recognition process. This is a widely observed phenomenon among both environmental and pathogenic species (Table 1).

Although common, the role of autoaggregation is in many cases poorly understood. The autoaggregative phenotype may be constitutive or induced under certain conditions, such as stress, oxygen availability or a change in temperature, depending on the bacteria in question [6]–[9]. As autoaggregation generally protects from external stresses, it can be beneficial for both environmental and pathogenic bacteria, particularly under conditions such as nutrient starvation or oxidative stress [10]–[12]. Autoaggregation and microcolony formation may also play a role in protection from the host immune system [13],[14]. Here, we review the current knowledge on autoaggregation, the role of autoaggregation in biofilm formation and pathogenesis, and molecular mechanisms leading to aggregation using specific examples.

### 1.2. A note on terminology

Several terms are used interchangeably for microbial self-aggregation, though these have subtle differences in meaning. “Autoaggregation” and “autoagglutination” are essentially synonymous. In both, the prefix “auto-” refers to self, i.e. only bacteria of the same strain bind together. “Aggregation” refers to the collection of particles into a single body and is, in our view, the clearest and most specific term and should therefore be preferred. The term “agglutination” originates from immunology, where visible aggregates are formed in a previously homogenous suspension upon the addition of an agglutinin, classically an antibody. Agglutination thus presupposes a crosslinking agent that binds suspended particles together. In the case of bacterial autoagglutination, the autoagglutinin would therefore refer to the surface molecule mediating the aggregation. However, agglutinins need not be bacterial molecules, as the example of antibody-mediated agglutination demonstrates. An example of non-bacterial agglutinins would be calcium and magnesium ions that cause aggregation of *Escherichia coli* cells lacking abundant outer membrane proteins [15].

“Flocculation” is another term often encountered when describing bacterial aggregation, particularly in environmental settings. The strict definition of flocculation is the formation of aggregates, either spontaneously or by a flocculating agent, that precipitate out of suspension. As flocculation is a description of a phenomenon rather than a mechanism, and can occur between different bacterial species, we prefer to use the more exact term “autoaggregation”. The prosaic term “clumping”, though also often used to describe bacterial aggregation, is similarly vague. Thus, we recommend using “autoaggregation” to describe the formation of aggregates of a single bacterial strain.

### 1.3. Classes of autoagglutinins

Autoagglutinins, by definition, mediate autoaggregation through homotypic interactions. Molecules of several different classes can act as autoagglutinins (Table 1). Autoaggregation is generally mediated by surface proteins. In some cases, also carbohydrates, particularly exopolysaccharides, can act as autoagglutinins. An example of an exopolysaccharide agglutinin is the polysaccharide intercellular adhesin (poly-N-acetylglucosamine; PNAG) of staphylococci [16]. A different example of carbohydrate-mediated autoaggregation is found in *Campylobacter jejuni*, where the autoaggregative phenotype is dependent on glycosylation of flagella [17]. Extracellular DNA (eDNA), which is often part of biofilm matrices, can also act as an agglutinin [18],[19].

**Table 1.** Examples of autoaggregating bacteria and their autoagglutinins.

Organism	Lifestyle	Autoagglutinin	Molecular class	Reference
*Acinetobacter baumannii*	Environmental bacterium and opportunistic pathogen	AtaA	TAA	[20]
*Actinobacillus pleuropneumoniae*	Respiratory pathogen of swine	Apa	TAA	[21]
*Aggregatibacter actinomycetemcomitans*	Periodontal pathogen	Flp	Type IV pilus	[22]
*Bartonella henselae*	Vector-born pathogen (cat scratch disease)	BadA	TAA	[23]
*Bartonella quintana*	Vector-born pathogen (trench fever)	VompA	TAA	[24]
*Bordetella pertussis*	Respiratory pathogen	FHA	TpsA (TVbSS)	[25]
*Burkholderia cenocepacia*	Environmental bacterium, opportunistic pathogen especially of CF patients	Cbl	C-U pilus	[26]
*Burkholderia pseudomallei*	Systemic pathogen (melioidosis)	Pil	Type IV pilus	[27]
*Campylobacter jejuni*	Gastrointestinal pathogen	FlaA (with glycosylation)	Flagellin protein	[28]
*Cronobacter sakazakii*	Opportunistic nosocomial and foodborne pathogen	FliC	Flagellin protein	[29]
*Escherichia coli*	Gastrointestinal commensal/pathogen	AAF/I	C-U pilus	[30]
		AIDA-I	SAAT	[31]
		Ag43	SAAT	[32]
		Bfp	Type IV pilus	[33]
		EibD	TAA	[34]
		Hek	Hra family β-barrel	[35]
		Hra1	Hra family β-barrel	[36]
		TibA	SAAT	[37]
*Edwardsiella tarda*	Fish pathogen	EseB	Type 3 secretion system translocator protein	[38]
*Haemophilus influenzae*	Respiratory pathogen	Hap	SAAT	[39]
*Moraxella catarrhalis*	Respiratory pathogen	MID (Hag)	TAA	[40]
*Lactobacillus plantarum*	Lactic acid bacterium	D1	LysM-containing serine/threonine-rich protein	[41]
*Legionella pneumophila*	Waterborne pathogen	Lcl	Collagen-like protein	[42]
*Myxococcus xanthus*	Social predatory bacterium	Pil	Type IV pilus	[43]
*Neisseria gonorrhoeae*	Sexually transmitted pathogen	Pil	Type IV pilus	[44]
*Neisseria meningitidis*	Nasopharyngeal opportunistic pathogen	AutA	SAAT	[19]
		Pil	Type IV pilus	[45]
*Pseudomonas aeruginosa*	Opportunistic pathogen, especially of CF patient lungs	PAK	Type IV pilus	[46]
*Rhizobium leguminosarum*	Symbiotic nitrogen-fixing bacterium	RapA1	Rap family protein	[47]
*Salmonella enterica*	Gastrointestinal pathogen	SE17	Curli	[48]
*Sinorhizobium meliloti*	Symbiotic nitrogen-fixing bacterium	EPS II	Exopolysaccharide	[1]
*Staphylococcus aureus*	Nasopharyngeal opportunistic pathogen	SasG	MSCRAMM	[49]
		PNAG	Exopolysaccharide	[16]
*Staphylococcus epidermidis*	Skin opportunistic pathogen	Aap	MSCRAMM	[50]
		PNAG	Exopolysaccharide	[51]
*Streptococcus pyogenes*	Respiratory pathogen	M1	M protein	[52]
*Vibrio cholerae*	Gastrointestinal pathogen	TCP	Type IV pilus	[53]
*Yersinia enterocolitica*	Gastrointestinal pathogen	MRHA	C-U pilus	[54]
		YadA	TAA	[55]
*Yersinia pestis*	Systemic pathogen (plague)	Ail (OmpX)	OmpX family β-barrel	[56]
		YPO0502	HCP	[57]
		YapC	SAAT	[58]
*Xanthomonas campestris*	Plant pathogen	Fim	Type IV pilus	[59]

Proteinaceous autoagglutinins include pili and fimbriae [30],[46], flagella [28],[29], large adhesin proteins such as M proteins and MSCRAMMs (for microbial surface components recognising adhesive matrix molecules) [49],[52], and small β-barrel proteins [36],[56]. Pili and fimbriae are found in both Gram-positive and Gram-negative organisms. These are long, fibrous structures composed of multiple subunits. These include pili assembled by the chaperone-usher (C-U) pathway in Gram-negative bacteria [60], curli fibres [61], and contractile type IV pili [62]. M proteins of *Streptococcus pyogenes* and MSCRAMMS of staphylococci are large single-chain polypeptides anchored to the cell wall [63],[64]. Small β-barrel proteins such as Hra1 from *E. coli* or Ail from *Yersinia pestis* are small (<30 kDa) integral transmembrane proteins of the outer membranes of Gram-negative cells. These proteins consist of a β-barrel transmembrane domain, the loops of which extend into the extracellular space and can thus interact with loops from proteins on the surface of a neighbouring bacterium [65]. Examples are listed in Table 1, and we describe selected examples in detail in later sections.

A class of proteins that is particularly rich in autoagglutinins is the autotransporter family of Gram-negative bacteria [66]. Autotransporters comprise the type V secretion system and are classed into five subtypes, including classical autotransporters (type Va), two-partner secretion systems (type Vb), trimeric autotransporter adhesins (type Vc), patatin-like autotransporters (type Vd), and inverse autotransporters (type Ve) [67]. With the exception of the poorly studied type Vd systems, all autotransporter classes include autoagglutinins [25],[32],[34],[68]. Among these, particularly the self-associating autotransporters (SAATs) of the *Enterobacteriaceae* (representing type Va) [69] and many type Vc secreted-proteins, such as the *Yersinia* adhesin YadA, BadA from *Bartonella henselae* and AtaA from certain strains of *Acinetobacter baumannii* [20],[23],[70], promote strong autoaggregation.

## 2. Measuring autoaggregation

**Figure 1.**
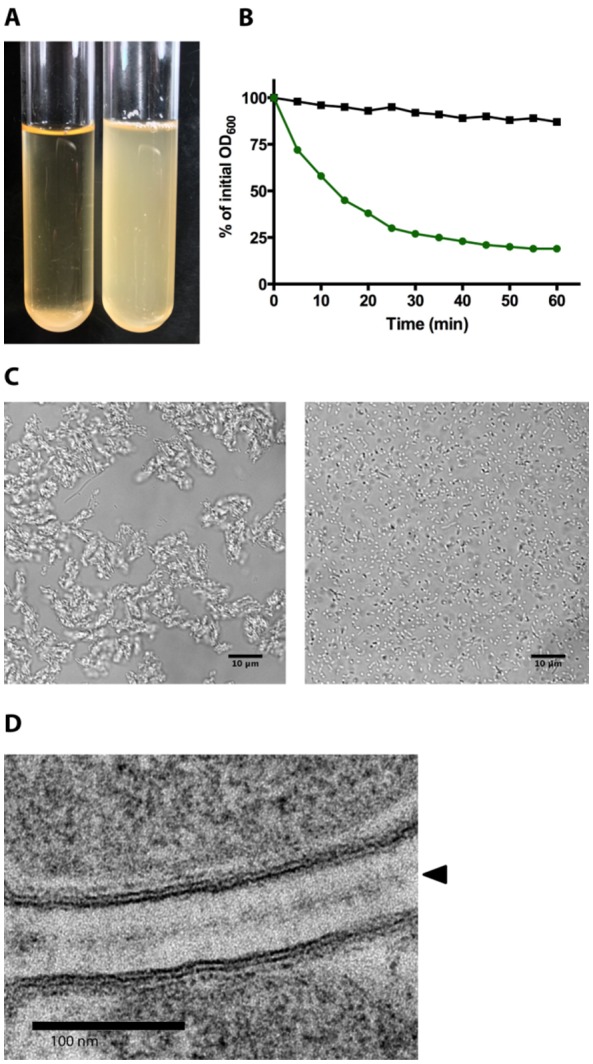
Measuring autoaggregation. (A) Macroscopic analysis of autoaggregation. *E. coli* cells expressing YadA (left tube) aggregate and settle at the bottom of the culture tube under static incubation, whereas an empty vector control culture (right tube) remains turbid. (B) Illustration of a sedimentation assay. The simplest way to measure aggregation quantitatively is to perform a sedimentation assay. Cultures are incubated statically, and periodically the OD_600_ value at the top of the culture tube is measured. In this illustration, the reduction in turbidity at the top of the culture is given as a percentage of the initial OD_600_ value. Autoaggregating bacteria settle at the bottom of the tube, resulting in a loss of turbidity (green curve), whereas in control cultures the reduction in turbidity is less pronounced (black curve). (C) Microscopic analysis of autoaggregation using phase contrast microscopy. Control cells (right micrograph) remain single, whereas YadA-expressing bacteria clump and form tightly packed aggregates (left micrograph). (D) Ultrastructural analysis of autoaggregation. Transmission electron micrograph of YadA-expressing bacteria. The lollipop-shaped YadA molecules interact through their head domains, keeping the cells at a uniform distance from each other. The interacting head domains in the centre of the space between the cells give rise to a zipper-like structure (arrowhead). The micrograph was kindly provided by Nandini Chauhan (University of Oslo, Norway) and Matthias Flötenmayer (Max Planck Institute for Developmental Biology, Tübingen, Germany).

There are several ways of demonstrating autoaggregation in bacteria. The simplest is to let cultures stand statically in narrow culture tubes for a given time and photograph the results (Figure 1A). Control cultures remain turbid, whereas autoaggregating cultures will settle at the bottom of the tube. The time required for observing sedimentation this way varies depending on the agglutinins and bacteria present, from a few minutes to several hours to overnight [15],[21],[28],[36]. The time window for observing differential aggregation behaviour compared with controls may have to be optimised; particularly in the case of non-motile bacteria, as also non-aggregating strains will eventually settle at the bottom of tubes due to gravity.

For more quantitative analysis, autoaggregation is usually measured by a sedimentation or settling assay [40],[71]–[73]. The set-up is similar to the one described above: the sedimentation of aggregates is recorded by measuring the turbidity of the cultures from the top of the tubes at given intervals (Figure 1B). The reduction in turbidity is then plotted as a function of time, either as the value of the optical density or as the fraction of the initial turbidity. Alternatively, the fraction of aggregating cells can be given as the complement of the residual turbidity in the supernatant (i.e. the fraction of the turbidity in the supernatant subtracted from unity) [5],[74].

This type of assay is often called an “autoaggregation” or “autoagglutination” assay, but formally it should be called a sedimentation assay. The assay should only be called an autoaggregation assay if the aggregation is measured in real time, i.e. beginning with the formation of flocs upon induction of the autoagglutinin. If performed this way, the assay usually takes longer as the aggregates must first form before they begin to precipitate. Often, the formation of aggregates causes an initial increase in apparent turbidity, as the forming flocs scatter light more strongly than individual cells. In most cases, sedimentation assays measure aggregation that has already taken place. Aggregates form in shaken cultures and once these are incubated statically, the aggregates begin to precipitate. The kinetics of the sedimentation is therefore faster than in a *bona fide* aggregation assay, and there is generally no initial increase in the turbidity. The rate of aggregation can be derived from sedimentation data and expressed as the change in turbidity (usually read as the optical density at 600 nm) per minute [35]. Another method for quantifying autoaggregation is comparing the turbidity of a static culture to a vortexed control culture. The ratio of the turbidity of the static culture to the vortexed culture is then plotted [65].

Over the past few years, flow cytometry has also been increasingly employed to investigate bacterial autoaggregation [7],[26],[75]. Flow cytometry is a method for analysing the physical properties of particles between approximately 1 and 100 µm in size, where the particles (e.g. single bacteria or bacterial aggregates) suspended in a fluid stream are passed through detectors one by one [76]. Scattered light gives an indication of the size of the particle, cell, or aggregate, whereas fluorescence can be used to differentiate between different bacterial subpopulations based on e.g. reporter gene expression.

Autoaggregation can also be observed microscopically (Figure 1C). The distribution of cells in a sample allows more parameters to be checked, such as aggregate size or the average number of cells in aggregates. Using differentially labelled cells, e.g. by expressing different fluorescent proteins in different populations, allows for the examination of aggregation behaviour between different strains by fluorescence microscopy [31],[77]. Ultrastructural analysis by electron microscopy can give further information, e.g. on the distance between aggregating cells [29],[34],[44],[78] (Figure 1D).

## 3. Molecular models for autoaggregation

The molecular mechanisms underlying bacterial autoaggregation vary. The mechanism can be simple surface electrostatic effects, e.g. by cells aggregating due to hydrophobic surface properties in an aqueous solution [6],[71],[79],[80]. Also bacteria with a charged surface may aggregate in the presence of an oppositely charged agglutinin, for example positively charged meningococci aggregating in the presence of eDNA, which is a polyanion [81]. Further, non-adsorbing polymers may cause bacteria to autoaggregate through depletion interactions [82]. However, in most cases bacterial autoaggregation is mediated by homotypic interactions between surface proteins.

A number of proteins are known to mediate autoaggregation (Table 1), but the molecular mechanisms of the interaction have only been determined in a handful of cases. Self-association motifs or residues have been found in some proteins, such as the outer membrane proteins Hek from *E. coli* [65] and Ail from *Y. pestis* [83]. However, how these motifs self-interact is not clear. In the case of TAAs, electron microscopy shows the sticky, globular head domains of the lollipop-like molecules interacting in a zipper-like fashion (Figure 1D) [34],[40],[78],[84]. The abundance of these proteins on the cell surface is high enough to coat the entire cell and the consequent interactions between TAAs on two cells strong enough to rip off outer membranes [34].

**Figure 2.**
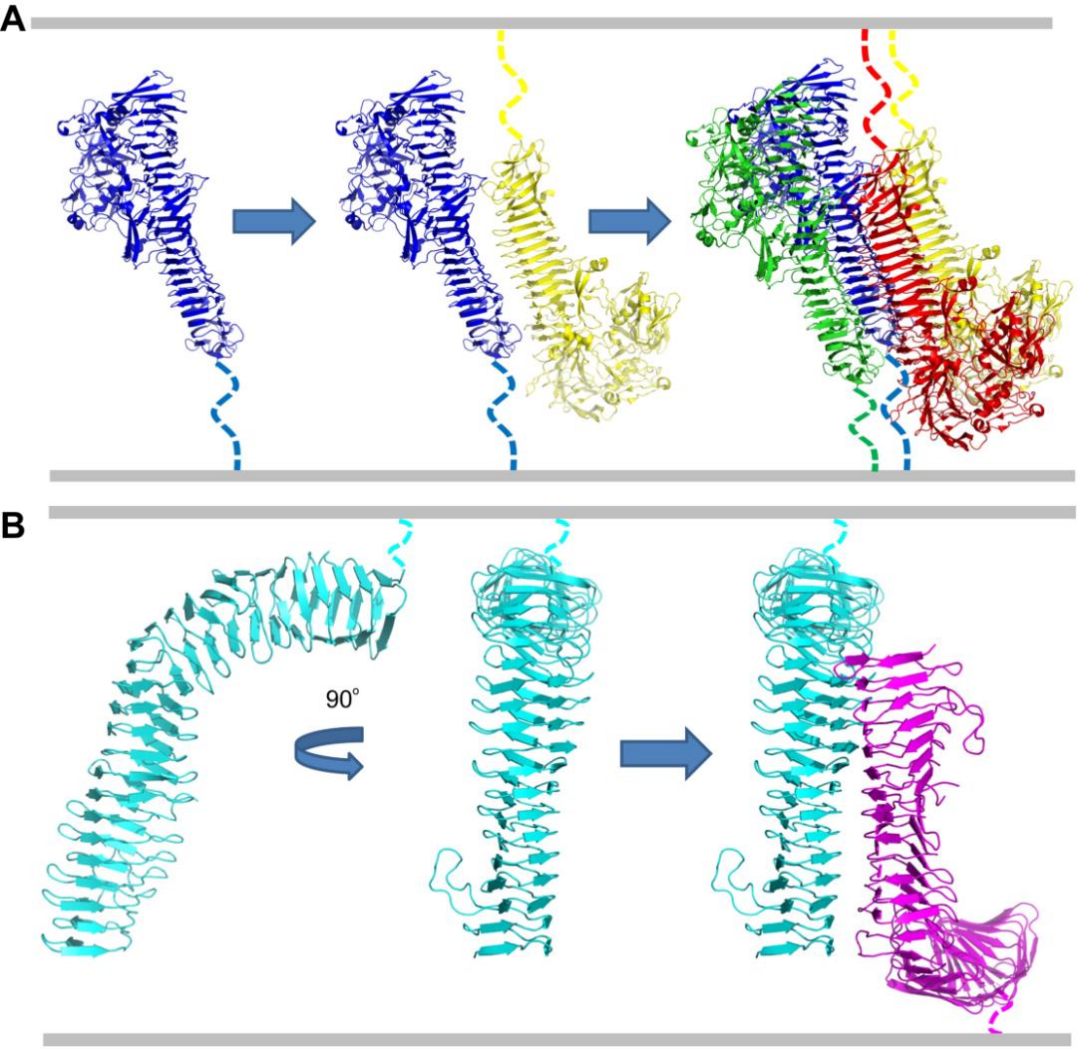
Molecular models for autoaggregation in self-associating autotransporters. (A) Model for self-association of the *Haemophilus influenzae* autotransporter Hap. First two Hap monomers (in blue and yellow) interact in a *trans* orientation. This allows a second *trans*-dimer (green and red) to be recruited to the complex. Additional dimers are added in an iterative fashion to stabilise the autoaggregation interface. The dotted lines denote the connection to the outer membrane (not part of the crystal structure). The grey bars show the approximate positions of the outer membranes of two neighbouring bacteria. The model is based on the Hap crystal structure (PDB ID: 3SYJ) [85]. (B) Model for self-association of the *E. coli* autotransporter Ag43. Two Ag43 monomers (cyan and magenta) interact via the “stalk” of the L-shaped molecules. The dotted lines denote the connection to the outer membrane (not part of the crystal structure). The grey bars show the approximate positions of the outer membranes of two neighbouring bacteria. The model is based on the Ag43 crystal structure (PDB: 4KH3) [77]. The structures are not to scale.

The crystal structures of two SAATs have shed light on the molecular mechanism underlying autoaggregation mediated by these proteins. The extracellular region of the *Haemophilus influenzae* autotransporter adhesin Hap resembles a “Dane axe”, with a protruding protease domain at the N-terminus (the “axe blade”) and a β-helical stalk (the “handle”) at the C-terminus (Figure 2A) [85]. The C-terminal region of Hap harbours the autoaggregative function [86]. This β-helical region forms a straight, triangular structure, with the edges having a hydrophilic, stacked Asn/Asp ladder [85]. In the Hap crystal structure, the Asn/Asp ladder of the edge of one Hap forms contacts with the F2 face of a second Hap molecule in a *trans* configuration (Figure 2A). This in turn creates an interface that can recruit more Hap molecules, which results in a huge increase of buried surface area (>7000 Å^2^ for the tetramer; Figure 2A). The interfaces are such that the recruitment of even further Hap dimers is possible, leading to a densely packed multimeric complex. It should be noted that mutating residues in the Asn/Asp ladder to alanine does not significantly reduce the self-aggregating properties of Hap. Rather than through direct hydrogen bonding, Meng et al. suggest that the aggregation is mediated by van der Waals forces derived from self-complementary interacting surfaces [85]. Thus, multimer formation should be a low affinity interaction and entropy-driven. This model is supported by dynamic light scattering data, showing strongly temperature-dependent polymerisation [85]. Soluble Hap monomers in dilute solution do not aggregate. As Hap can be cleaved from the cell surface, this allows for a mechanism whereby the aggregation interface can be depolymerised to allow bacteria to escape from microcolonies or biofilm [87].

In contrast to Hap, Antigen 43 (Ag43) from *E. coli* self-associates through a polar interaction network, with both hydrogen bonds and salt bridges [77]. The Ag43 extracellular region forms an L-shaped β-helix (Figure 2B). The self-association interface resides in the “stem” of the L, with a ladder-like configuration of interacting residues reminiscent of Hap. Also like Hap, the interaction is in a *trans* orientation. The L-shape of Ag43 is also important for autoaggregation, as mutations straightening the β-helical spine of the protein abolished the ability to autoaggregate [77].

## 4. Autoaggregation and biofilms

Biofilm can be defined as a surface-attached community of bacterial cells embedded in a self-produced polymeric matrix [88],[89]. Biofilms can form on both biotic and abiotic surfaces, and in addition floating biofilms (referred to as pellicles) can form at liquid-air interfaces [90]. The formation of biofilm occurs when bacteria switch from a planktonic state to a surface-attached state, and it occurs in multiple stages starting from the initial attachment followed by microcolony and macrocolony formation. In final stages after the mature biofilm has formed, bacteria detach and become free-swimming again for dispersal. In the environment, biofilms are the major form of bacterial growth [91]. Biofilms also play an important role in many diseases [92]. The biofilm environment provides protection against a number of stresses, and bacteria within biofilms can be up to 1,000-fold more resistant towards antibiotics [93].

**Figure 3.**
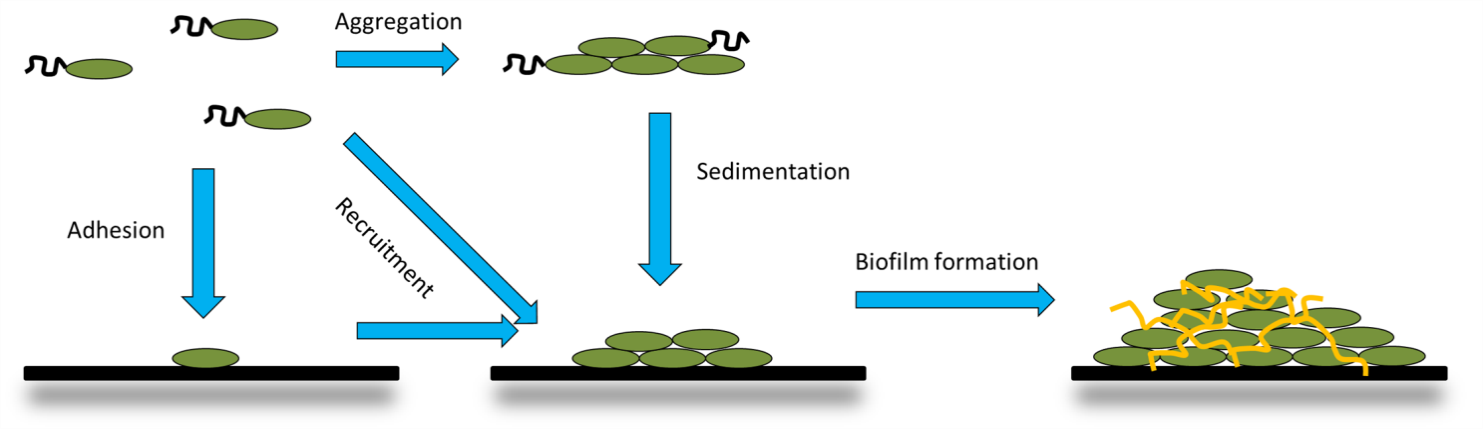
The role of autoaggregation in biofilm formation. Autoaggregation can lead to biofilm formation in two ways: planktonic bacteria can either attach to a substrate surface as single cells and then recruit more planktonic cells via aggregation to form a single microcolony, or planktonic cells aggregate in suspension and then settle on the substrate surface. Both pathways can lead to the formation of biofilm.

Autoaggregation and microcolony formation are among the first steps in building a biofilm. Autoaggregation can lead to microcolony formation and biofilm in two ways (Figure 3). In the first, single planktonic cells attach to the substrate. This depends on expression of surface adhesins and possibly also motility factors [46]. Following this, these cells recruit other cells from suspension via autoagglutinins, leading to microcolony formation [75],[94]. This is sometimes referred to as co-adhesion [95]. Alternatively, single cells can migrate along the surface, e.g. using type IV pili, and aggregate together [46],[94]. In the other mechanism, cells autoaggregate in solution, and the aggregates settle on the substrate to initiate biofilm formation [2]. These two mechanisms may be simultaneously at play. At high cell densities, aggregated cells have a competitive advantage over single cells, as the cells positioned at the top of the aggregate have more access to nutrients. However, at low cell densities, the aggregated cells are at a disadvantage, as the cells in the middle of the aggregate have limited nutrient access [2]. The shape of the aggregate is also predicted to affect competition: rounded aggregates fare better at higher cell densities, whereas more spread aggregates that maximise surface area have an advantage when competition is low [96].

Biofilm formation is often mediated by quorum sensing. Quorum sensing is a cell density-dependent system for regulating bacterial collective behaviour [97]. In *E. coli*, quorum sensing mediated by the autoinducer-2 (AI-2) molecule also promotes autoaggregation. AI-2 is a chemotactic signal for *E. coli* that promotes motile cells to seek out each other and then aggregate via Ag43 or curli, which in turn leads to biofilm formation [98]. Chemotaxis also plays a role in the autoaggregation of *Azospirillum brasiliense*, a plant-associated soil bacterium [99]. Here, the chemotactic signal transduction pathway Che1 increases swimming velocity with changes in aeration conditions; mutants defective in Che1 do not alter their swimming speed and do not detach from early, reversibly formed clumps. This in turn leads to formation of larger flocs stabilised by exopolysaccharides [100] These examples demonstrate that autoaggregation is not always simply a passive phenomenon but can be an active process, where cells expend energy to move along chemoattractant gradients to join forming aggregates.

In contrast to the examples above, autoaggregation does not always promote biofilm formation. An example against the general trend is *Bordetella holmesii*, where the protein BipA acts as an anti-agglutination factor that promotes biofilm formation [101]. In the absence of BipA, *B. holmesii* failed to form biofilm despite strong autoaggregation. Another example is *Burkholderia pseudomallei*, where microcolony formation is mediated by type IV pili in a temperature-regulated manner [27]. However, in some *B. pseudomallei* strains, type IV pili are not needed for biofilm development, and in some conditions the lack of type IV pili can increase biofilm formation. Thus, microcolony and biofilm formation are two separate processes [27].

## 5. Autoaggregation in environmental bacteria

Autoaggregation has been observed in a variety of environmental species, including isolates from drinking water, activated sludge, fermented foods, and industrial and intestinal sources [10],[20],[41],[74],[102]. Suspended bacterial aggregates can offer some of the same benefits and protection as biofilm; it is therefore no surprise that the switch from planktonic to aggregated growth is triggered under conditions of environmental stress, be it toxins, antibiotics, predation, or lack of nutrients [3],[103],[104]. However, in contrast to the sessile bacteria in biofilms, suspended aggregates can maintain their mobility [11].

### 5.1. Aggregation in response to chemical stress

Species of the genus *Pseudomonas*, belonging to the γ-proteobacterial phylum, can survive and thrive in a broad range of environments, partly due to a high capacity to endure both endogenous and exogenous stresses [105]. *P. putida* CP1 is capable of degrading chloroaromatic compounds such as the isomers 2-chlorophenol, 3-chlorophenol and 4-chlorophenol [6]. The degradation of all three monochlorophenols proceeds through an *ortho*-cleavage pathway. High concentrations of these chloroaromatics resulted in autoaggregation of cells in the culture medium, whereas no cell aggregation could be observed using lower concentrations during growth. This suggests that cell aggregation resulted from the toxic effects of monochlorophenols at higher concentrations. This conclusion was supported by the fact that *P. putida* CP1 grown on high concentrations of phenol, which is more easily degraded than monochlorophenols and thus confers less chemical stress, showed no aggregation, as well as by an increase in autoaggregation observed in relation with increasing toxicity of the different monochlorophenol isomers. Thus, autoaggregation in *P. putida* CP1 occurs as a result of chemical stress and is connected to chlorophenol removal at higher substrate concentrations, suggesting a protective advantage of autoaggregation, allowing chlorophenol degradation to occur [6].

The active formation of cell aggregates is also a stress response in *P. aeruginosa* and serves as a survival mechanism when exposed to certain detergents such as sodium dodecyl sulfate (SDS) [106],[107]. The genes *siaA* (SDS-induced-aggregation A) and *siaD* are essential for induction of autoaggregation as a specific response to SDS and have been suggested to be responsible for perceiving and transducing SDS-related stress [108]. SiaA encodes a putative membrane protein harboring a HAMP (histidine kinases, adenylyl cyclases, methyl-accepting chemotaxis proteins and phosphatases) domain and a PP2C (Protein phosphatase 2C)-like phosphatase domain. Both domains are essential in two-component signal transduction [109],[110]. SiaA is believed to be a stress sensor and important for signal transduction as a response to an environmental stimulus. SiaD is predicted to encode a putative cytoplasmic di-guanylate cyclase involved in the biosynthesis of cyclic di-guanosine monophosphate (c-di-GMP), thus suggesting that SDS-induced aggregation is regulated through a c-di-GMP-dependent signal transduction pathway [108]. Thus, autoaggregation in *P. aeruginosa* can increase fitness under unstable and potentially harmful environmental conditions for suspended cells.

### 5.2. Aggregation in response to predation

Another important feature of autoaggregation is the defense against predators. When *Pseudomonas* sp. MWH1 was cultured in the presence of the bacterivorous flagellate *Ochromonas*, formation of floc-like, suspended microcolonies of up to a 1,000 cells conferred protection against flagellate grazing, whereas bacteria cultured in the absence of predation grew as planktonic cells. Thus, autoaggregation is a survival strategy under strong grazing pressure [104]. Also *Sphingobium* sp. Z007 formed aggregates in co-cultures with the bacteriovorous protozoan *Poterioochromonas*, and supernatants from such a culture could induce aggregation of *Sphingobium* in a monoculture, suggesting the presence of soluble signaling molecules released into the supernatant in response to predation [103]. Similarly, *P. aeruginosa* formed microcolonies that conferred protection against grazing in the presence of a predatory protozoan. Microcolony formation depended on type IV pili, flagella and alginate production [111]. It is possible that *Pseudomonas* species employ similar survival mechanisms when it comes to the prevention of phagocytosis by cells of the immune system of the host.

### 5.3. Bacterial co-aggregation

Co-aggregation describes the formation of bacterial aggregates among different bacterial species. In a simplified model system of a naturally occurring aquatic bacterial community, the effects of sub-lethal concentrations of antibiotics were tested. The model system contained four bacterial species: *Aeromonas hydrophila*, *Brevundimonas intermedia*, *Micrococcus luteus* and *Rhodococcus* sp. Upon exposure to antibiotics, a decrease in bacterial fitness was observed accompanied by a reduction in bacterial cell numbers by ∼75% [3]. The bacterial community switched rapidly from a planktonic lifestyle to forming microcolonies as well as larger co-aggregates in the presence of antibiotics, and the bacteria were able to maintain their viability in the aggregative state. Bacteria organized in these aggregates were surrounded by a self-synthesized exopolymeric matrix, thus creating a microenvironment where, through conspecific and interspecific interactions, general resistance to antibiotics increased [3]. Thus, autoaggregation and co-aggregation help in the fast adaptation to antibiotics in aquatic systems resulting in an enhanced survival rate of bacterial cells. Although in no direct correlation with pathogenicity of these bacterial species, this study shows yet another example of aggregation-dependent tolerance against antimicrobial resistance which could be a mechanism to evade host defenses during bacterial infection. Consistent with this, co-aggregation of oral bacteria increased resistance to phagocytosis and promoted abscess formation in a mouse subcutaneous infection model [4]. Co-aggregation may also play a role in the formation of late-stage dental biofilms. The species *Veillonella atypica* co-aggregates with a significant number of potential dental pathogens via its Hag1 TAA [112]. *Veillonella* may thus act as a “bridging species” by recruiting late-stage, potentially pathogenic species such as *Porphyromonas gingivalis* to the forming dental biofilm.

Different bacterial species can also co-aggregate due to predation pressure. When cultured together, the bacterial species *Arthrobacter agilis* and *Brevundimonas* sp. GC044 competed with each other, with *A. agilis* reaching only 2% of the total cell density. In contrast, when these bacteria were co-cultured in the presence of the predatory protozoan *Poterioochromonas*, the proportion of *A. agilis* rose to 6–10% [113]. In co-culture with no predation, the bacteria grew mostly as single cells, but under predation conditions they formed increased amounts of either single-species microcolonies of a few cells or larger aggregates containing both species, and the total number of cells was higher than in monocultures with predation. However, the predator also grew to significantly higher densities in bacterial co-cultures compared to monocultures, and a larger proportion of predators were found attached to aggregates in the co-cultures. This suggests that co-aggregation is not simply an anti-grazing mechanism. The observations that the biomass in the grazed co-cultures was substantially higher than in the corresponding monocultures, and that more dissolved organic matter was transferred to the predator, suggest that co-aggregation and emerging interactions in such a complex microbial communities increase the overall efficiency of the ecosystem [113].

## 6. Autoaggregation and pathogenesis

Autoaggregation is often associated with pathogenesis, although in most cases the direct effect of autoaggregation remains unresolved. Autoaggregation-dependent microcolony formation could result in an effective increase in concentration of secreted effectors at or near the host cells that modulates virulence [17]. Aggregation has also been shown to influence pathogenesis by increasing tolerance against antimicrobial agents [3],[11],[114], elevating invasion frequency as well as invasion efficiency of host cells [42], impeding phagocytosis by cells of the host immune system [14], or increasing survival within phagosomes [13]. Autoaggregation has been frequently observed in pathogenic bacteria (see Table 1), and may contribute to virulence by promoting bacterial survival and fitness in general. Thus, autoaggregation can have a beneficial but passive effect on pathogenesis by providing a growth advantage as well as a microenvironment for undisturbed bacterial growth protected from otherwise harsh environmental conditions or host defenses. This results in prolonged bacterial persistence within the host and an enhanced chance of successful colonization and invasion. Below, we review the effects of autoaggregation relevant to pathogenesis in selected bacteria.

### 6.1. Escherichia coli

*E. coli* are Gram-negative bacteria belonging to the phylum γ-*Proteobacteria* with a wide range of both commensal and pathogenic strains. The *flu*-encoded autotransporter protein Ag43 is expressed by a high percentage of enteropathogenic (EPEC) and uropathogenic *E. coli* strains [115] and belongs to the family of autotransporter proteins. Ag43 is a SAAT and Ag43-mediated aggregation provides a mechanism for reducing local oxygen concentrations, thereby conferring a high protection against oxidizing agents and H_2_O_2_ killing. However, Ag43 expression does not appear to be directly linked to H_2_O_2_-induced stress and Ag43 mediated protection against H_2_O_2_-killing is believed to be a side effect of Ag43 mediated aggregation [8]. Further, Ag43-mediated autoaggregation protected the bacteria against killing by neutrophils, although the aggregated cells were more efficiently phagocytosed [13]. Ag43 of uropathogenic strains was also shown to contribute to long-term persistence of bacteria in the bladder, possibly by enhancement of biofilm formation following initial autoaggregation [116]. However, it was reported that disruption of the *flu* gene had no influence on the interaction of the UPEC IH11128 Dr^+^ strain (*dra*^+^; *dra* operon-encoded genes are essential for the biogenesis of the adhesive Dr fimbriae) with host receptors in the first step of host invasion by this bacterium, showing that Ag43 does not act as a specific adhesin or invasin. Nonetheless, internalized UPEC IH11128 Dr^+^ Ag43^+^ cells were viable after 72 h post infection, whereas only 7% of UPEC IH11128 Dr^+^ Ag43^−^ survived the first 24 hours post infection, and no viable cells could be detected after 48 hours post infection. Thus, Ag43 is believed to enhance intracellular survival and virulence due to the formation of intracellular aggregates [117].

In addition to Ag43, different *E. coli* pathotypes produce a variety of related autotransporter autoagglutinins, many of which have virulence-associated properties such as binding to and invading host cells [118]. Pathogenic *E. coli* also produce several TAA autoagglutinins involved in pathogenesis [119]–[122]. Among other classes of autoagglutinins, EPEC express type IV pili called bundle-forming pili (Bfp) that are involved in binding to host cells and necessary for full virulence of EPEC [33]. Bfp plays a major role in EPEC microcolony formation, which in turn leads to biofilm formation [123]. However, twitching motility conferred by Bfp is also required for bacterial dispersal from microcolonies. A mutant defective in Bfp contraction was 200-fold less virulent than the wild-type; thus, Bfp-mediated twitching motility is also required for full virulence [33].

### 6.2. Legionella pneumophila

*Legionella pneumophila* is a Gram-negative, facultative intracellular microorganism [124] belonging to the γ-*Proteobacteria* and a major cause of community-acquired pneumonia [125]. The natural hosts of *L. pneumophila* are amoebae and replication occurs within the host after phagocytosis. The ability to form autoaggregates increases the ability of *L. pneumophila* to come in contact with its host and can potentiate the infection of the hosts as shown in *Acanthamoeba castellanii* infection experiments. Host internalization of *L. pneumophila* was eight times greater for cell aggregates compared to planktonic cells. Also, Lcl (Legionella collagen-like protein)-dependent autoaggregation increased the number of *L. pneumophila* bacteria per infected *A. castellanii* cell and required the presence of divalent cations [42]. Lcl is involved in both biofilm production and adherence to human cells [126]. Thus, autoaggregation can enhance virulence by means of an increased invasion frequency as well as efficiency. This has also been shown in the case of *Bartonella henselae* [127].

### 6.3. Pseudomonas aeruginosa

*P. aeruginosa* is an opportunistic pathogen and a major agent in nosocomial infections. *P. aeruginosa* is regarded as a global health problem due to its intrinsic resistance to a wide range of antibiotics and the lack of a vaccine [128]. *P. aeruginosa* is a major pathogen in cystic fibrosis (CF) lung disease. Since the late 1980s, aggressive antibiotic treatment is applied after positive diagnosis for *P. aeruginosa* in CF-patients, resulting in a significant postponement of chronic *P. aeruginosa* infection [129]. Planktonic bacteria are most vulnerable to eradication by host defenses and antibiotic treatment in the early stages of a CF infection. One of the earliest symptoms detectable already in infants with CF is an accumulation of neutrophils and neutrophil-derived products in the bronchoalveolar lavage fluid of the patients [130]. The formation of aggregates by *P. aeruginosa* is enhanced by human neutrophils [114],[131] and an early-stage neutrophil-induced aggregation confers antibiotic resistance and selective upregulation of quorum sensing signaling resulting in an enhanced virulence. The acquired antibiotic resistance was lost upon DNAse treatment and the consequent disintegration of the aggregates, showing a direct correlation between autoaggregation and antimicrobial resistance [114].

*Pseudomonas aeruginosa* isolates from single CF patients exhibit a number of growth phenotypes, including a small colony variant (SCV) [132]. This variant is highly autoaggregative and resistant to antibiotics and arises at high frequency *in vivo*, but can revert back to an antibiotic-sensitive, wild-type phenotype [133]. Several SCV isolates were more hydrophobic than wild-type *P. aeruginosa* and were hyperpiliated with type IV pili, which presumably contribute to the aggregative phenotype [134]. However, C-U pili have also been suggested to be responsible for the SCV autoaggregative phenotype [132].

### 6.4. Staphylococcus aureus

The opportunistic human pathogen *Staphylococcus aureus* is a Gram-positive bacterium associated with a wide range of diseases, not least CF lung disease [135]. As mentioned above, CF patients are subject to an aggressive antimicrobial treatment from the early stages of the disease. As in the case of *P. aeruginosa*, cell aggregation of *S. aureus* confers a tolerance to various antibiotics [11] and can even be a result of antibiotic treatment in the first place [136]. Due to the fact that many of those antibiotics have different cellular targets, ranging from protein synthesis to DNA replication and cell wall biosynthesis, as well as the fact that protection could be completely abolished by disruption of the aggregates, the protective mechanism is believed to be the consequence of the physical barrier provided by autoaggregation [11]. Haaber et al. could also show that, in contrast to biofilms, cell aggregates of *S. aureus* showed an on average 7-fold higher metabolic activity than in planktonic cells. Cells from aggregates kept this high metabolic level even after disruption of the aggregates via sonication. Similarly to data from biofilms, a slightly increased mutation frequency was observed in cells grown in aggregates compared to planktonic cells [11]. Thus, autoaggregation is believed to provide bacteria with the benefits of biofilm while maintaining mobility resulting in an advanced evasion advantage from host defenses and antimicrobial treatment.

### 6.5. Yersinia spp.

The genus *Yersinia* is a member of the family *Enterobacteriaceae* and consists of 18 species [137],[138], including three human pathogens among the otherwise environmental, avirulent species. All three pathogenic species are invasive: *Y. enterocolitica* and *Y. pseudotuberculosis* cause gastrointestinal illness and more rarely systemic infections, whereas *Y. pestis* is the causative agent of plague [139].

Autoaggregation is common in pathogenic *Yersiniae* [140],[141] and has been used for decades as a quick method to identify pathogenic strains [142]–[144]. *Yersinia* strains show strong autoaggregation when cultured at 37 °C, whereas avirulent strains lack the autoaggregation phenotype [140]. Kapperud & Lassen observed autoaggregation in about 70% of human and animal clinical isolates of *Y. enterocolitica*, whereas all environmental isolates tested in their study were negative for autoaggregation [141].

Several key players aid in the formation of aggregates in *Yersiniae*. The *Yersinia* adhesin YadA is involved in autoaggregation [9] and is crucial for the packing density of microcolonies observed in *Y. enterocolitica* in collagen gels [145]. YadA is a central virulence factor of *Y. enterocolitica* and, in addition to autoaggregation, mediates binding to host cells and extracellular matrix components, evasion of phagocytosis, and serum resistance [139]. The autoaggregation function of YadA has been used to demonstrate surface display of the extracellular domain, where mutations within the β-barrel domain of the protein impeded secretion of the lollipop-like extracellular region [146],[147]. YadA is expressed at mammalian body temperatures and is exclusively responsible for autoaggregation in *Y. enterocolitica* and *Y. pseudotuberculosis* grown at 37 °C [148]. At lower temperatures, the mannose-resistant hemagglutinin (MRHA), a C-U-assembled pilus, mediates autoaggregation of *Y. enterocolitica* strains [54].

The Ail (Attachment and Invasion Locus; also called OmpX) protein of *Y. pestis* is a small outer membrane β-barrel protein that has orthologues in both *Y. enterocolitica* and *Y. pseudotuberculosis*. Ail is involved in adherence and internalization into epithelial host cells and also mediates autoaggregation in *Y. pestis* [56]. Ail-mediated autoaggregation was observed at both 28 °C and 37 °C, but the resulting flocs were larger at 28 °C. In addition to Ail, other factors implicated in *Y. pestis* autoaggregation have been identified. One is YPO0502, which belongs to the family of hemolysin co-regulated proteins (HCPs) that are secreted by type VI secretion systems [57]. YPO0502 was extracted from autoaggregating *Y. pestis* grown at 26 °C. Another *Y. pestis* protein that mediates autoaggregation when expressed in *E. coli* is the autotransporter YapC [58]. However, deleting the *yapC* gene did not yield an altered autoaggregative phenotype in *Y. pestis* [149].

Another key player identified in the formation of *Y. pestis* cell aggregates appears to be phosphoglucomutase (PgmA), which is required for efficient autoaggregation and plays an important role in antimicrobial peptide resistance [149]. PgmA converts glucose-6-phosphate into glucose-1-phosphate, which is a precursor for surface-exposed carbohydrate-containing structures including lipopolysaccharide (LPS). However, the LPS structure of *pgmA* mutant of *Y. pestis* was not altered [149]; thus, PgmA must exert its effect through some other glycosylated molecule, the identity of which remains to be elucidated.

## 7. Conclusions

Autoaggregation, though clearly a widespread and important phenomenon, is poorly understood. Although several lines of evidence show that bacteria are more protected from environmental or immunological stresses in the aggregated state, in many cases detailed experiments have not—or cannot—be performed to study the exact effects of autoaggregation on bacterial growth or survival under adverse conditions. For many bacteria, investigating autoaggregation is hampered by the fact that the autoagglutinin(s) involved is unknown. Another confounding factor is that, when known, many autoagglutinins are multifunctional proteins. One problem with assessing the effect of autoaggregation in virulence is the difficulty in finding point mutations that abolish autoaggregation without affecting other functions of the protein in question. Thus, for multifunctional proteins such as YadA, the exact role of autoaggregation has not been addressed due to lack of a tractable system where the other activities of YadA, such as collagen binding or serum resistance, would not be compromised. In some cases, such as Ag43, a number of point mutations are required to prevent autoaggregation [77]. Therefore, one of the hurdles that must be overcome to investigate the role of autoaggregation specifically is to be able to find systems where the autoaggregative function can be uncoupled from other activities of the agglutinin. A third confounding factor is that a single bacterial species may elaborate a number of agglutinins, as demonstrated by *Y. pestis* (see section 6.4). Therefore, studying the effects of one autoagglutinin may require deleting the others as well, which in turn raises the question of how physiologically relevant such an experimental set up might be.

Though known for several decades now, the phenomenon of aggregation has produced hardly any applications. However, one possible application may be the neutralisation of pathogenic strains by co-aggregation with probiotic bacteria [150]. In the aggregated state, pathogens, especially of the gastro-intestinal and urinary tracts, would not be able to reach the mucosal surface to colonise the host. This may actually be one of the mechanisms of how some probiotic strains exert their beneficial effects, though it has not been widely realised [151]. Future probiotics might be engineered to include autoagglutinins from multiple major pathogens to render invading pathogens less virulent and thus reduce the risk of infection.

As a final note, autoaggregation may also play a role in competition between bacteria. Autoaggregation, by definition, can only take place between closely related bacteria. In this sense, it could be considered a form of kin selection. This view is supported by the recent work showing that, under high competition with single cells, cells positioned at the top of aggregates enjoy a competitive advantage [2]. However, this comes at the expense of the cells at the bottom and centre of the aggregate, who must effectively forgo replication in favour of their kin cells positioned more advantageously. Large, spherical aggregates would thus be favoured only in the situation where all the bacteria are related [96]. The autoagglutinins mediating autoaggregation would therefore be under diversifying selection in order to be able to distinguish kin from non-kin. If considered in this light, autoaggregation should perhaps be grouped with type VI secretion and contact-dependent growth inhibition systems, and bacteriocins as a (less belligerent) bacterial competition mechanism. More studies need to be carried out to fully delineate the role of autoaggregation in inter-strain or inter-species competition, protection from the environment and bacterial virulence.
